# Influence of early through late fusion on pancreas segmentation from imperfectly registered multimodal magnetic resonance imaging

**DOI:** 10.1117/1.JMI.12.2.024008

**Published:** 2025-04-26

**Authors:** Lucas W. Remedios, Han Liu, Samuel W. Remedios, Lianrui Zuo, Adam M. Saunders, Shunxing Bao, Yuankai Huo, Alvin C. Powers, John Virostko, Bennett A. Landman

**Affiliations:** a Vanderbilt University, Department of Computer Science, Nashville, Tennessee, United States; b Johns Hopkins University, Department of Computer Science, Baltimore, Maryland, United States; c National Institutes of Health, Department of Radiology and Imaging Sciences, Bethesda, Maryland, United States; d Vanderbilt University, Department of Electrical and Computer Engineering, Nashville, Tennessee, United States; e Vanderbilt University Medical Center, Department of Medicine, Division of Diabetes, Endocrinology, and Metabolism, Nashville, Tennessee, United States; f VA Tennessee Valley Healthcare System, Nashville, Tennessee, United States; g Vanderbilt University, Department of Molecular Physiology and Biophysics, Nashville, Tennessee, United States; h University of Texas at Austin, Dell Medical School, Department of Diagnostic Medicine, Austin, Texas, United States; i University of Texas at Austin, Livestrong Cancer Institutes, Dell Medical School, Austin, Texas, United States; j University of Texas at Austin, Department of Oncology, Dell Medical School, Austin, Texas, United States; k University of Texas at Austin, Oden Institute for Computational Engineering and Sciences, Austin, Texas, United States; l Vanderbilt University, Department of Biomedical Engineering, Nashville, Tennessee, United States

**Keywords:** multimodal, fusion, pancreas segmentation, registration, magnetic resonance imaging, UNet, nnUNet

## Abstract

**Purpose:**

Combining different types of medical imaging data, through multimodal fusion, promises better segmentation of anatomical structures, such as the pancreas. Strategic implementation of multimodal fusion could improve our ability to study diseases such as diabetes. However, where to perform fusion in deep learning models is still an open question. It is unclear if there is a single best location to fuse information when analyzing pairs of imperfectly aligned images or if the optimal fusion location depends on the specific model being used. Two main challenges when using multiple imaging modalities to study the pancreas are (1) the pancreas and surrounding abdominal anatomy have a deformable structure, making it difficult to consistently align the images and (2) breathing by the individual during image collection further complicates the alignment between multimodal images. Even after using state-of-the-art deformable image registration techniques, specifically designed to align abdominal images, multimodal images of the abdomen are often not perfectly aligned. We examine how the choice of different fusion points, ranging from early in the image processing pipeline to later stages, impacts the segmentation of the pancreas on imperfectly registered multimodal magnetic resonance (MR) images.

**Approach:**

Our dataset consists of 353 pairs of T2-weighted (T2w) and T1-weighted (T1w) abdominal MR images from 163 subjects with accompanying pancreas segmentation labels drawn mainly based on the T2w images. Because the T2w images were acquired in an interleaved manner across two breath holds and the T1w images on one breath hold, there were three different breath holds impacting the alignment of each pair of images. We used deeds, a state-of-the-art deformable abdominal image registration method to align the image pairs. Then, we trained a collection of basic UNets with different fusion points, spanning from early to late layers in the model, to assess how early through late fusion influenced segmentation performance on imperfectly aligned images. To investigate whether performance differences on key fusion points are generalized to other architectures, we expanded our experiments to nnUNet.

**Results:**

The single-modality T2w baseline using a basic UNet model had a median Dice score of 0.766, whereas the same baseline on the nnUNet model achieved 0.824. For each fusion approach, we analyzed the differences in performance with Dice residuals, by subtracting the baseline score from the fusion score for each datapoint. For the basic UNet, the best fusion approach was from early/mid fusion and occurred in the middle of the encoder with a median Dice residual of +0.012 compared with the baseline. For the nnUNet, the best fusion approach was early fusion through naïve image concatenation before the model, with a median Dice residual of +0.004 compared with the baseline. After Bonferroni correction, the distributions of the Dice scores for these best fusion approaches were found to be statistically significant (p<0.05) via the paired Wilcoxon signed-rank test against the baseline.

**Conclusions:**

Fusion in specific blocks can improve performance, but the best blocks for fusion are model-specific, and the gains are small. In imperfectly registered datasets, fusion is a nuanced problem, with the art of design remaining vital for uncovering potential insights. Future innovation is needed to better address fusion in cases of imperfect alignment of abdominal image pairs. The code associated with this project is available here https://github.com/MASILab/influence_of_fusion_on_pancreas_segmentation.

## 1 Introduction

Multimodal fusion is the combination of information from different modalities with the aim of improving understanding of a scene, such as combining information from natural images and infrared images.^1^ In medical imaging, multimodal fusion combines information from multiple modalities, including imaging and tabular data, to provide a more complete assessment of the subject condition based on complementary information.^2^^,^^3^ In pure multimodal medical image fusion, combining imaging modalities that extract different anatomical information of the same subject is a rational approach, such as leveraging both computed tomography images for the bone, and magnetic resonance (MR) images for soft tissue characterization.^4^ In some medical imaging problems, it can be useful to combine several types of MR images, as has been shown in the brain tumor segmentation challenge (BraTS), where T1w, contrast-enhanced T1w, T2w, and fluid-attenuated recovery T2w MR images were fused to provide a more complete analysis of brain tumors.^5^

In this work, the disease of interest is diabetes, which greatly affects patients’ lives and can lead to complications such as vision impairment, kidney disease, and even lower limb amputation.^6^^,^^7^ Diabetes is a global health crisis, with an estimated global prevalence of 10.5% in 2021.^8^ The pancreas has two major compartments, endocrine and exocrine, with the insulin-secreting pancreatic islets of the endocrine compartment comprising about 2% of the pancreas volume. Pancreatic islet dysfunction plays a major role in most forms of diabetes.^9^ The total pancreatic volume is reduced in type 1 diabetes,^10^^–^^13^ indicating that the exocrine compartment is also altered. For these reasons, segmentation of the pancreatic images is relevant to understanding the changes in the pancreas in diabetes. In addition, pancreas segmentation may be useful in the research of other diseases, such as pancreatic cancer.^14^

Unfortunately, the pancreas is notoriously difficult to segment due to its variable shape and lack of distinct boundaries in medical images.^15^^,^^16^ It is sensible to leverage multiple imaging modalities that provide complementary information about structures around the pancreas to improve pancreas segmentation. For example, leveraging T2w MR images, which delineate the stomach and filled intestines, and T1w MR images, which delineate the liver and empty intestines, is a reasonable approach for better determining the pancreas boundary.

**Fig. 1:**
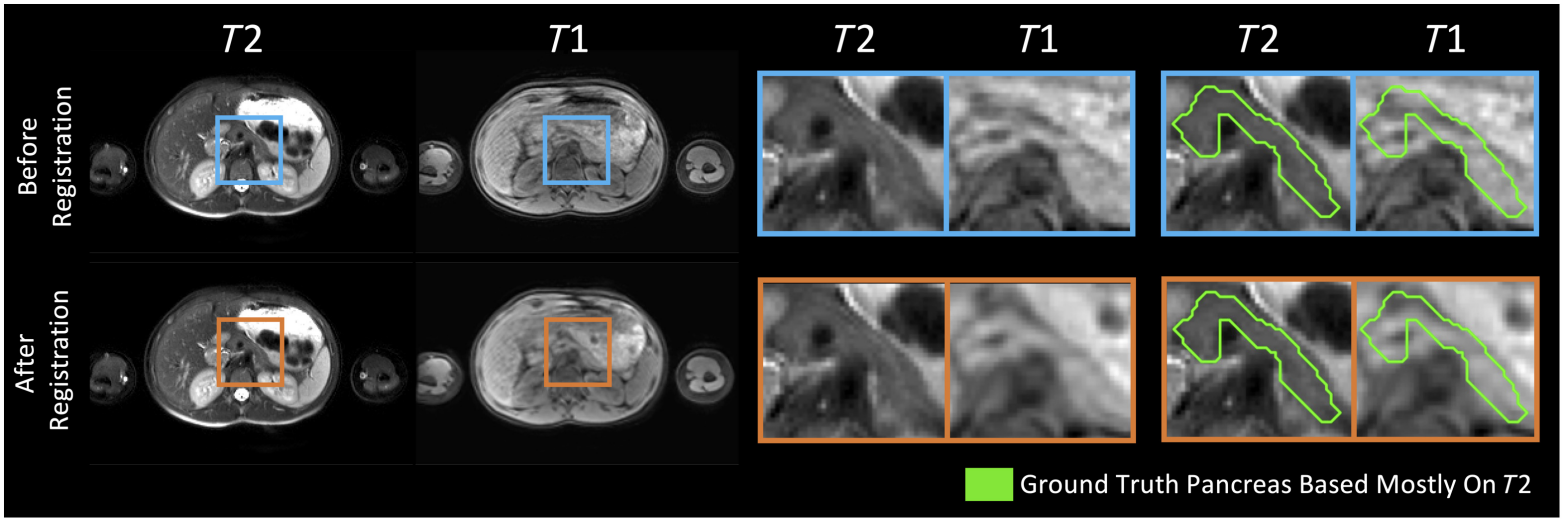
Our pancreas labels were mainly drawn based on the T2w MR images. Despite the use of effective deformable registration, the pancreas labels did not always line up well with the corresponding anatomy in the paired T1w MR images from the same subject and same imaging session. For example, the T1w image shown exhibits poor alignment with both the T2w image and the pancreas mask, even after registration (bottom right). Persisting image misalignment makes it unclear how best to fuse T1w information to see gains in segmentation performance. The example shown exhibits particularly poor alignment.

Generally, the first step in the fusion of multimodal medical images is registration so that the anatomical structures are aligned across modalities.^17^ In this work, we refer to paired scans that are not well aligned after registration, due to natural deformations of the abdomen, as imperfectly registered. In contrast to multimodal brain datasets, such as BraTS,^5^ abdominal imaging faces the challenge of flexible and highly deformable anatomy.^18^ Scans of the abdomen are often acquired by having subjects hold their breath to limit motion artifacts.^19^ Some approaches for scanning the abdomen use a single breath hold, whereas other techniques exist that use two breath holds, creating two images by skipping slices, and interleaving them to obtain a single image volume covering the whole abdomen. For example, in this manuscript, we have T2w abdominal images taken on two interleaved breath holds and paired T1w images taken on a single breath hold. Because of the deformable characteristics of abdominal imaging, achieving perfectly aligned multimodal data is difficult, even after effective deformable registration (Fig. 1).

Although terminology can vary in the literature, fusion of medical images can be generally separated into early fusion, late fusion, and hybrid fusion.^20^ These names are intuitive: early fusion means that a model combines multimodal information towards the beginning of processing, late fusion means information combination happens towards the end of processing, and hybrid fusion uses both early and late fusion.

Where in the deep learning model to perform multimodal medical image fusion is still an open question. Moreover, in the deformable anatomy of the abdomen, the best fusion approach for pancreas segmentation on multimodal MR images is unclear, especially because it is challenging to perfectly align anatomical structures across modalities. In response, we study (1) how the stage of fusion, from early through late, affects the quality of pancreas segmentation on imperfectly registered paired T2w and T1w MR images when using UNet^21^-based architectures and (2) whether there is a generalized “best” location to fuse in our problem space. To keep our approach straightforward, we focus on early through late fusion methods. As a result, we exclude hybrid fusion approaches, which can introduce additional complexity in interpretation.

## 2 Methods

**Fig. 2:**
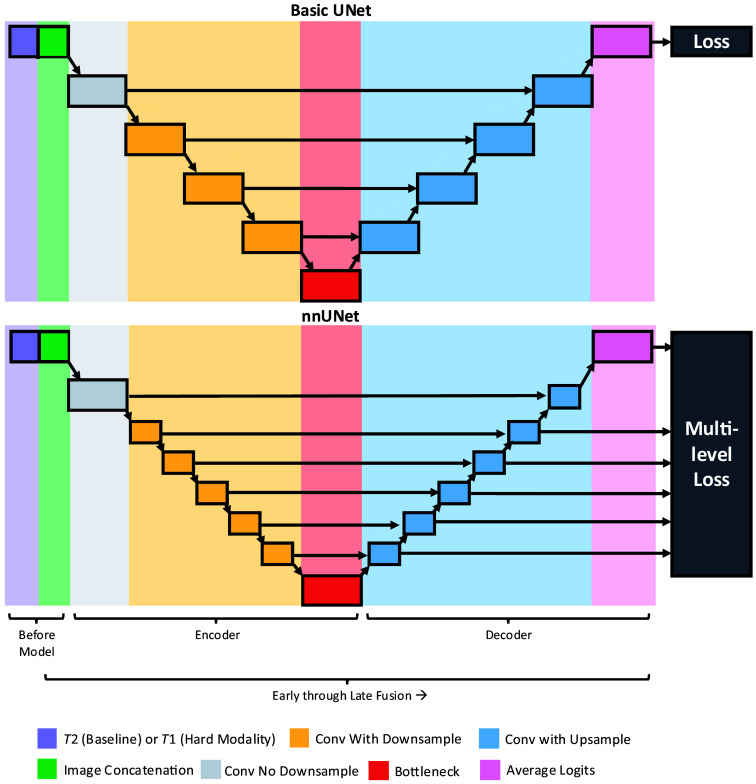
We studied how early through late fusion affects pancreas segmentation on imperfectly registered image pairs using UNet-based architectures. We used the MONAI basic UNet (top) and nnUNet (bottom) to investigate if there exists a generalizable “best” location to fuse for our problem domain. In this pursuit, we trained 13 basic UNet configurations and 17 nnUNet configurations, each with a single fusion point. All of the fusion points are shown above as colored blocks. All fusion was performed through concatenation, except for the latest fusion (pink), which used averaging logits.

We examined fusion at all main intermediate stages in UNet-based networks (Fig. 2). We first deformably registered the paired T2w and T1w abdominal MR images using deeds registration.^22^^,^^23^ Then, we trained a set of basic UNets^21^ with different fusion points to assess how early through late fusion influences pancreas segmentation on imperfectly registered data. We assessed whether our findings generalized to another model by validating on nnUNet.^24^ We selected nnUNet over other UNet variants due to its self-configuring nature based on the dataset, as well as its generalizable state-of-the-art performance.^24^

### 2.1 Data

Samples were studied in deidentified form from Vanderbilt University Medical Center under Institutional Review Board approval (IRB #130883) and are associated with ClinicalTrials.gov identifier: NCT03585153. After excluding scans with major motion artifacts, our dataset contained 353 image pairs of T2w and T1w abdominal MR images, which came from 163 subjects. Here, an image pair means a T1w and T2w image of the same subject acquired during the same imaging session. Each T2w image volume had a spatial resolution of 1.5  mm×1.5  mm×5  mm with a spatial dimension of 256×256×30  voxels. The T1w images were at a higher spatial resolution than the T2w images at [1.18,1.30]×[1.18,1.30]×2  mm with a spatial dimension of [240,320]×240×[75,98]  voxels. Each image pair had a corresponding manual delineation of the pancreas, which was performed using the T2w image except for cases where the T2w image had image artifacts precluding manual segmentation. In these cases (∼1%), the T1w image was used for segmentation. All the pancreas labels were drawn by a single experienced radiologist. Of the 163 subjects, 63 were controls, 66 had type 1 diabetes, 22 were autoantibody positive, and 12 had maturity-onset diabetes of the young (MODY). Most of this cohort were part of a previous study.^10^

### 2.2 Registration

The T2w/T1w image pairs were acquired during the same session. The T1w images were deformably registered to their paired T2w images using deed registration.^22^^,^^23^ Because the output of deeds is resampled to isotropic resolution by default, we resampled the isotropic T1w image back into the original T2w image space. Each registered image pair was qualitatively assessed for alignment between the T2w image, T1w image, and the pancreas mask. This quality assurance was performed by manually examining the pancreas overlays on the images as well as a checkerboard grid of the paired T2w and T1w images. Image pairs with particularly poor alignment were excluded from the study. The registered images were generally well-aligned; however, the registration was not perfect (Fig. 1).

### 2.3 UNet Fusion Configurations

To assess how fusion location affects pancreas segmentation in the UNet-style architecture, we opted for a straightforward design. Each architecture variant had one fusion point—the blocks on which fusion occurred are shown in Fig. 2. At each major block, we had an architecture variant that fused through concatenation. In addition, we trained a multimodal model that accepted T2w and T1w images that were fused in image space before being fed to the model. To handle very late fusion, we built an architecture that fused information by averaging logits. We also used single modality models as baselines: one for only T2w images and another for only T1w images. These choices resulted in 13 model variants for the basic UNet from MONAI^25^ and 17 variants for nnUNet.

### 2.4 Cross-validation

For five-fold cross-validation, each split was performed at the subject level and always stratified across conditions: control, type 1 diabetes, autoantibody-positive, and MODY, to reduce bias. Each cross-validation fold was partitioned into three sets: training, validation, and test. The training and validation sets consisted of 90% and 10% of the non-test subjects for the fold, respectively. The selection of subjects per fold was random. The validation and testing data contained a single scan from each subject, whereas we allowed multiple scans per subject in the training data.

For the basic UNets, we performed cross-validation on each of the 13 fusion configurations. We trained each basic UNet architecture using the same approach. Each MR image that was loaded had the top 0.001% of pixel intensities clipped and was min-max normalized between zero and one based on its minimum and maximum voxel intensity. The training loop consisted of 16,000 batches using a batch size of two image volumes, Dice loss,^26^ the Adam optimizer,^27^ the one-cycle learning rate scheduler^28^ with a max learning rate of 0.001, and early stopping. The model learned after each batch and performed validation every 20 steps. Early stopping engaged if the average validation Dice loss did not improve after 500 batches. The weights were selected based on the validation step with the lowest validation loss.

For the nnUNets, we used the default five-fold cross-validation and weight selection approach from nnUNet version 2. We stipulated that the data in each fold and train/validation/test split matched with our basic UNet experiments.

### 2.5 Evaluation and Statistical Analysis

To assess overall segmentation quality, we selected the Dice score over other possible metrics, such as Hausdorff Distance, as it measures overlap rather than boundary-based differences. To determine how fusion approaches affected performance, we compared with the single modality T2w-only baseline. Dice scores for the approaches were aggregated across the five folds of cross-validation before comparison via concatenation. Statistical analysis was performed using the paired Wilcoxon signed-rank test to compare the distributions of Dice scores for each fusion approach with the T2w-only baseline.

We evaluated two models, the basic UNet and nnUNet, to address different questions: (1) whether fusion improves performance over the baseline (basic UNet) and (2) whether observed improvements generalize across architectures (nnUNet). Because these represent distinct hypothesis families, Bonferroni correction was applied separately for each model. For both models, six pairwise comparisons were made, and p-values were adjusted accordingly by multiplying each raw p-value by the number of comparisons (six). The Bonferroni-corrected p-values were evaluated at three thresholds: corrected p<0.05 (*), corrected p<0.01 (**), and corrected p<0.001 (***).

### 2.6 Technical Implementation

All basic UNet experiments were implemented using Pytorch^29^ 1.12.1 and were based on the basic UNet architecture from MONAI^25^ 1.0.0. All of the nnUNet experiments were performed and adapted from the nnUNet version 2 GitHub: https://github.com/MIC-DKFZ/nnUNet and used their provided environment. The models were trained on NVIDIA RTX A6000s and NVIDIA Quadro RTX 5000s.

## 3 Results

### 3.1 Performance Relative to Baseline

**Fig. 3:**
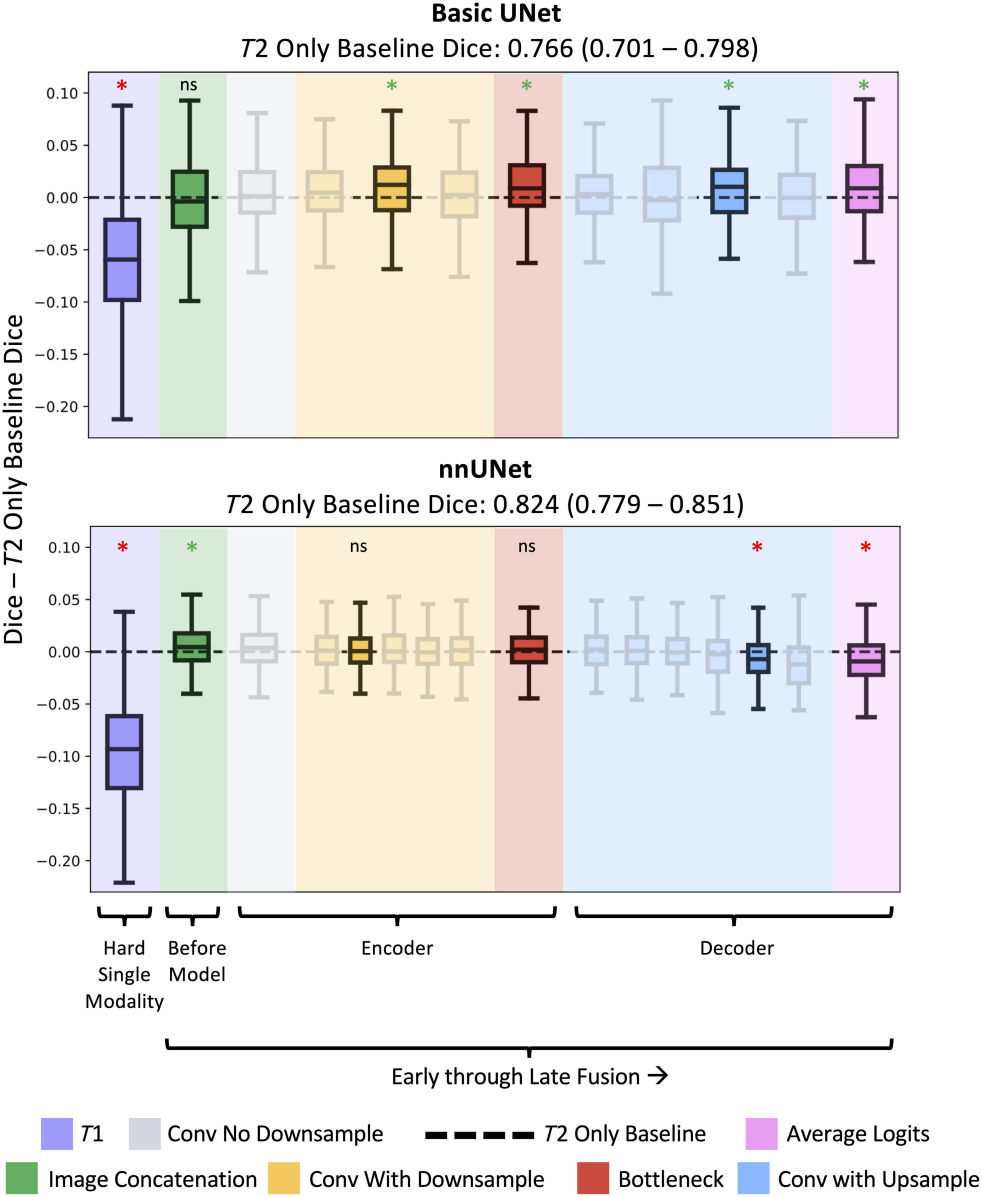
Fusion in specific blocks shows performance gains on both the basic UNet (top) and nnUNet (bottom), when compared with the T2w-only baseline (dotted line). However, these improved fusion points differ between the models and the gains are small. Key fusion points from the basic UNet are highlighted and transferred to nnUNet, as well as expected comparisons: T1w-only and naïve image concatenation. In the downsampling and upsampling portions of the models, correspondence between fusion points in the basic UNet and nnUNet was determined by counting i layers from either the beginning or end of the section. Asterisks denote statistically significant results from the Wilcoxon signed-rank test after Bonferroni correction, with green indicating improved performance and red indicating decreased performance. The models that are the focus of this figure are highlighted. All other fusion points are shown faded to summarize variability. Baseline values are provided as medians with interquartile range.

**Fig. 4:**
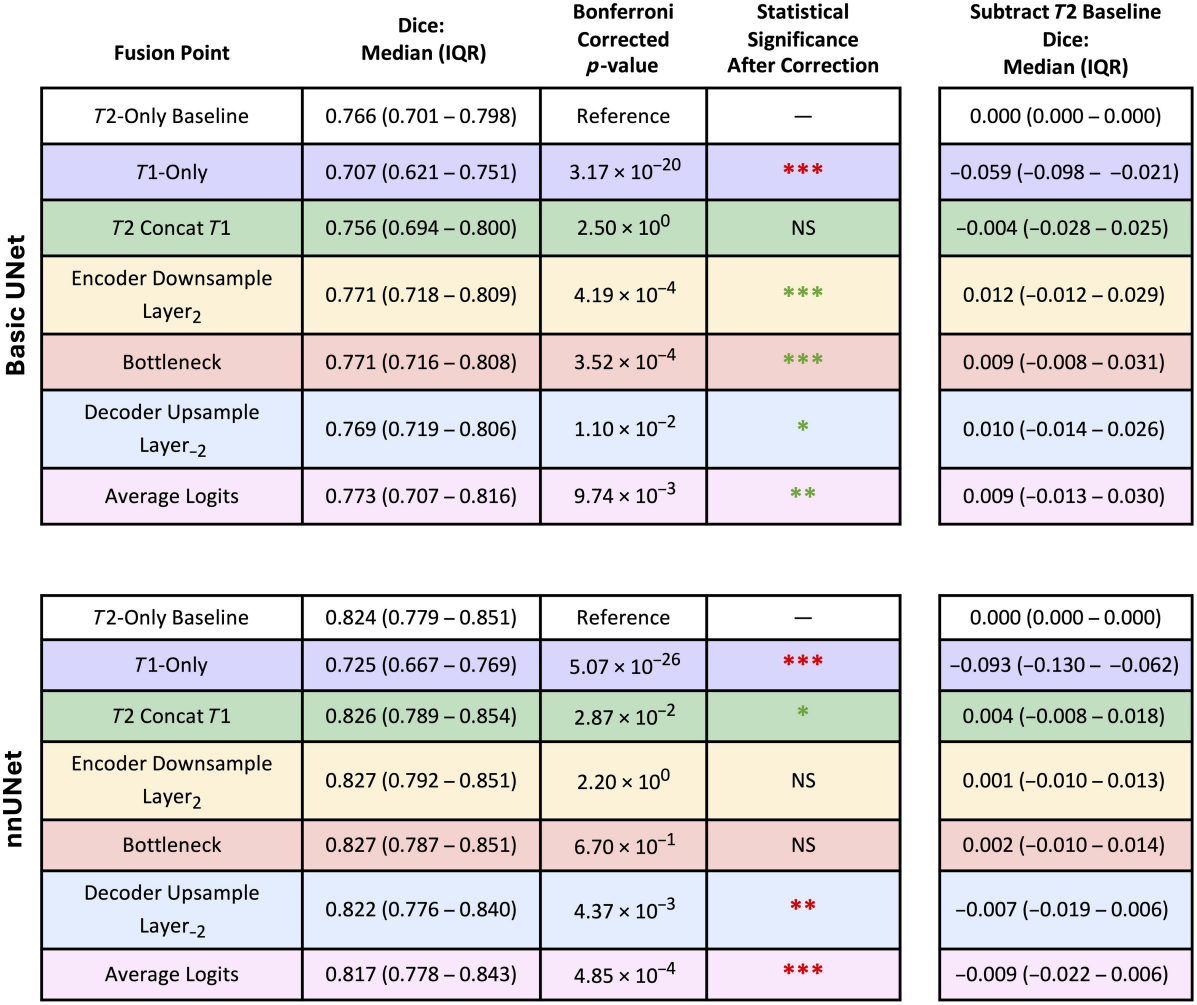
We complement Fig. 3 by providing numerical values for key fusion points while retaining the color coding. Statistical significance was set at α=0.05, with corrections applied to the p-values using the Bonferroni method. After Bonferroni correction, we indicate significance with asterisks: p<0.05 (*), p<0.01 (**), and p<0.001 (***). Green denotes performance increase and red denotes decrease. Compared with the basic UNet, nnUNet was less sensitive to incorporating T1w information. The nnUNet showed smaller changes in performance across fusion points but started with better baseline performance than the basic UNet. For the one fusion point on the nnUNet where performance improved and was statistically significant (T2 concat T1), the p-values for nnUNet were larger than those for the basic UNet during performance increases.

In Figs. 3 and 4, we examine performance for the basic UNet and nnUNet across all the trained approaches: the T2w-only baseline, the imperfectly registered T1w-only model, and the fusion configurations. Performance differences were small in terms of the Dice score,^30^ with the median of the best recorded improvement being ∼0.01 above the baseline. The T1w images were notably more challenging than the T2w images (Fig. 4). Difficulty on the T1w images alone was not surprising, because the labels were drawn mainly on the T2w images, and the T1w images were imperfectly registered. Fusion of the T2w and T1w modalities led to both improvements and reductions in performance compared with the baseline. Gains seen on key fusion points on the basic UNets did not transfer to nnUNet and vice versa. Improved performance from fusion on the basic UNet occurred in the middle of the encoder, at the bottleneck, in the middle of the decoder, and from the late fusion approach of averaging logits. The nnUNet showed improved performance from very early fusion through naïve image concatenation, with a steady decline in performance as fusion occurred later in the modeling process.

### 3.2 Sensitivity Analysis

**Fig. 5:**
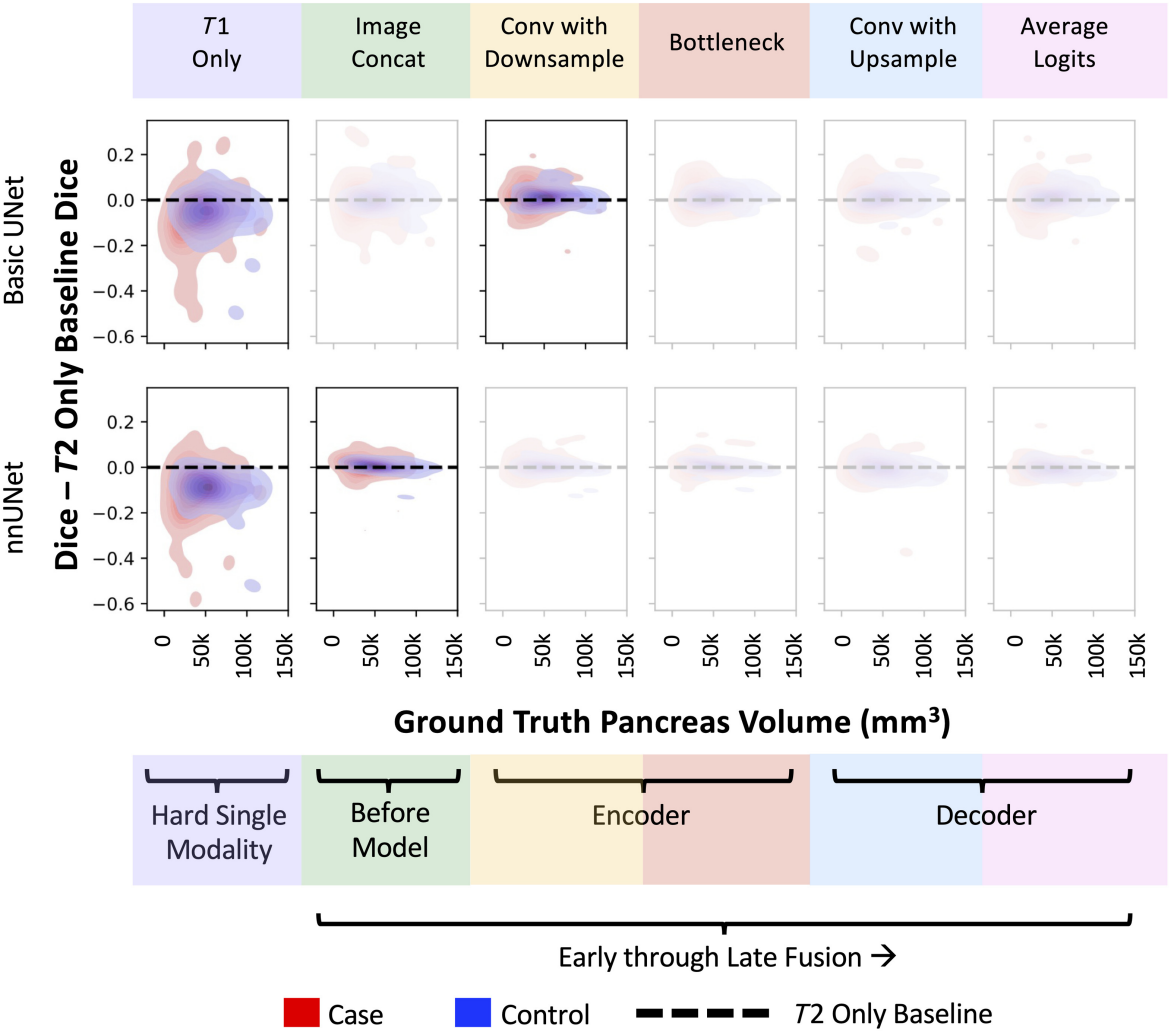
Generally, when our key fusion models performed better than the T2w-only baseline, it was more pronounced on smaller pancreases, which were usually from diabetic subjects (denoted as red contours). We highlight the T1w-only baseline in addition to the best fusion approach for both the basic UNet and nnUNet to demonstrate how fusing the more difficult T1w modality can improve performance compared with the T2w-only baseline (dotted line). Additional key fusion points are shown faded to summarize variability across fusion points.

**Fig. 6:**
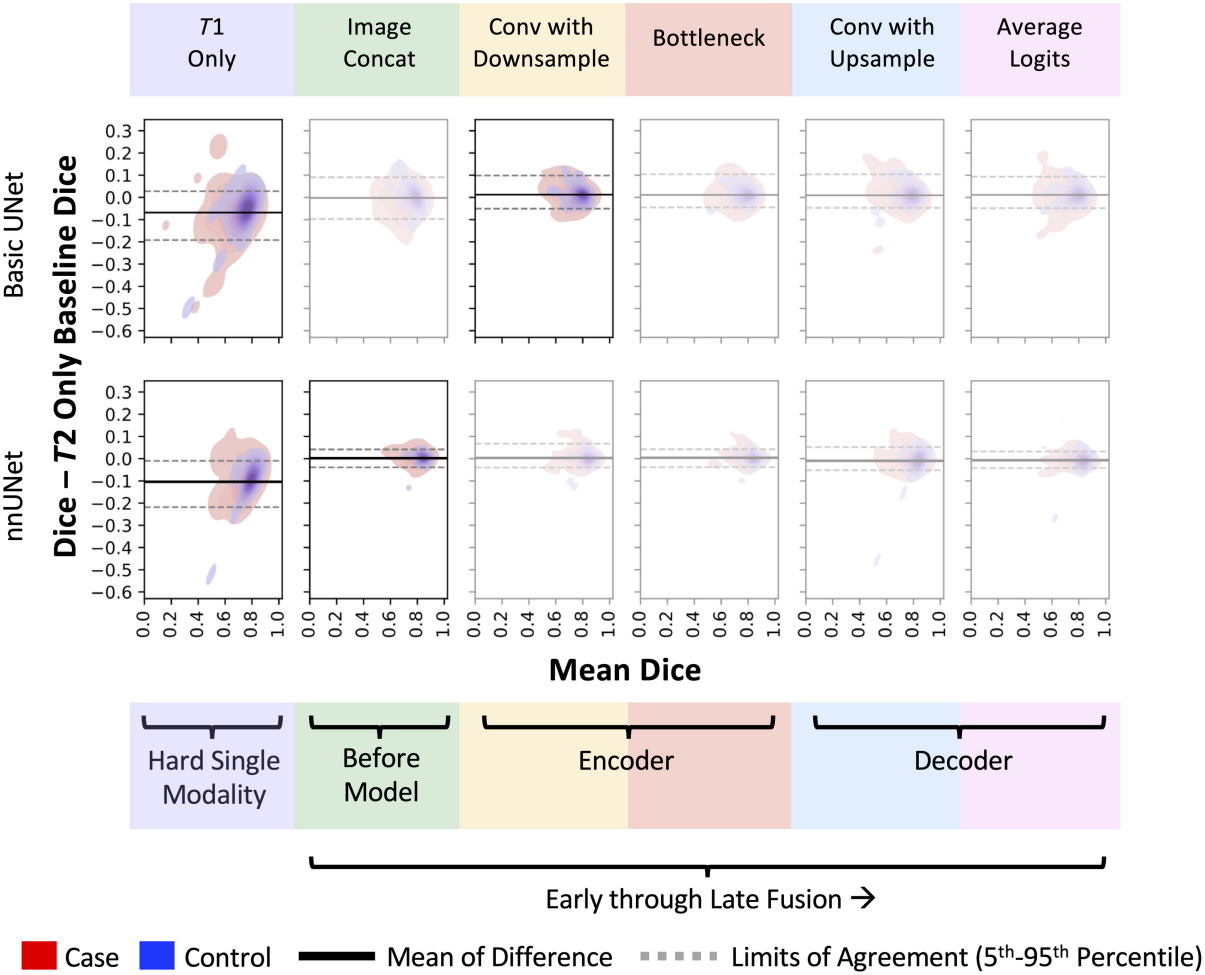
In this Bland Altman plot,^31^ we note that for the key fusion points (1) systemic biases were small and (2) the nnUNet configurations had greater agreement with the T2w-only baseline. The models that are the focus of this figure are highlighted: T1w only and the best configuration for each model. Additional key fusion points are shown faded to summarize variability. This Bland-Altman plot follows the nonparametric method recommendation by Bland and Altman.^32^ Consequently, the limits of agreement are represented as the 5th and 95th percentiles instead of the traditional mean ±1.96× standard deviation.

In Fig. 5, we show that when compared with the T2w-only baseline, the best fusion approaches generally improve performance on diabetic pancreases that are smaller in size. Performance gains occur despite the difficulty of identifying these same smaller pancreases when using the T1w images alone. In Fig. 6, we show that the systemic bias for fusion was small—the nnUNet models depicted more agreement with the baseline than the basic UNets.

### 3.3 Qualitative Results

**Fig. 7:**
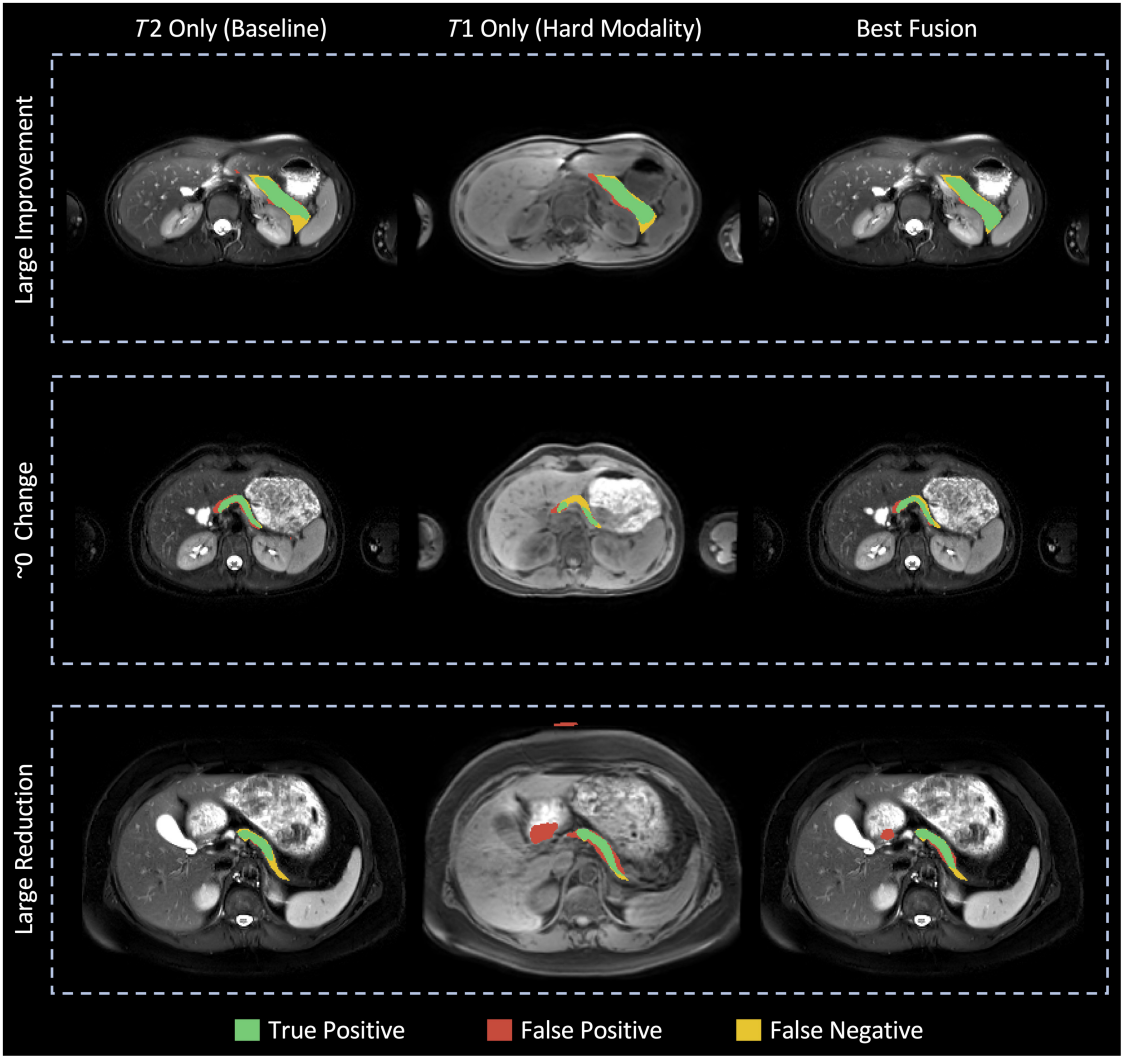
When the imperfectly registered T1w image was complementary to the T2w image, we saw improved performance through our best fusion (top row). If the T1w image did not complement the T2w image, fusion performance could be similar to the baseline (middle row). When the T1w image was challenging for segmentation, fusion could reduce performance (bottom row).

In Fig. 7, we visualized how the best fusion approach from the basic UNets compared with the T2w-only baseline and T1w-only model. By comparing the fusion to the single modality models, we noted three intuitive scenarios. When the T1w image provided a signal that assisted in pancreas segmentation, performance could improve. When the T1w image did not provide a helpful signal, the performance could be similar to the T2w-only baseline. When the T1w image presented information that made segmentation highly error-prone, fusion could reduce performance compared with the baseline.

## 4 Discussion and Conclusion

In this work, we designed experiments to discover if there were optimal and generalizable fusion points on UNet-based segmentation architectures when dealing with imperfectly registered multimodal data. The main finding was that improvements in fusion were small and model specific, rather than generalizable, even between the similar basic UNet and nnUNet architectures. These results indicate that further innovation may be needed to better capitalize on the complementary information in imperfectly registered image pairs.

## Data Availability

The code for this paper can be accessed at the following site: https://github.com/MASILab/influence_of_fusion_on_pancreas_segmentation. The data that support the findings of this article are not publicly available.
